# Cryo-EM structure of TGFBIp fibrils driven by a corneal dystrophy–linked mutation enables design of peptide inhibitors of aggregation

**DOI:** 10.1073/pnas.2607937123

**Published:** 2026-09-08

**Authors:** Yi Xiao Jiang, Lukasz Salwinski, Michael R. Sawaya, Peng Ge, Liisa Lutter, Xinyi Cheng, Carolyn J. Hu, Hillary Hernandez, David R. Boyer, Conrad Wang, Filipe A. Melo, Duilio Cascio, David S. Eisenberg

**Affiliations:** ^a^ Department of Biological Chemistry, University of California, Los Angeles, CA 90095; ^b^ Department of Chemistry and Biochemistry, University of California, Los Angeles, CA 90095; ^c^ Department of Energy Institute for Genomics and Proteomics, University of California, Los Angeles, CA 90095; ^d^ Molecular Biology Institute, University of California, Los Angeles, CA 90095; ^e^ Institute for Quantitative and Computational Biosciences, University of California, Los Angeles, CA 90095

**Keywords:** transforming growth factor β–induced protein, corneal dystrophy, amyloid fibril, peptide inhibitor, cryo-EM

## Abstract

Corneal dystrophy is a heterogeneous group of diseases which manifests clinically by progressive corneal opacity and diminishing visual acuity. A group of corneal dystrophies are linked to autosomal dominant mutations in transforming growth factor β–induced protein (TGFBIp) and characterized by extracellular amyloid-positive deposits of unknown molecular structure. Here, we determined the cryogenic-electron microscopy (cryo-EM) structure of amyloid fibrils formed by the TGFBIp FAS1-4 domain with corneal dystrophy–linked mutation V624M. The L569 to N609 fibril core, which includes the Y571-R588 segment enriched in patient corneal deposits, forms symmetrical protofilaments with internal solvent channels. Leveraging this structure, we designed peptide inhibitors intended to bind onto fibril ends to block elongation, targeting the unequal growth of symmetrical protofilaments. Our G1 and H4 inhibitors exhibit concentration-dependent reduction of TGFBIp FAS1-4 aggregation as assessed by Thioflavin T, solubility fractionation, and electron microscopy. Our work illustrates how fibril structures can guide rational inhibitor design and suggests the targeting of protein aggregates as a therapeutic approach for corneal and ocular diseases.

## Significance

Understanding the molecular basis of protein aggregation disorders is essential to developing treatments. One such disease is corneal dystrophy, genetically and pathologically associated with transforming growth factor β–induced protein (TGFBIp). Hitherto, molecular structures of TGFBIp aggregates are underexplored and no pharmacological treatment is available for this condition. We describe an unusual “inside-out” cryogenic-electron microscopy (cryo-EM) structure of fibrils formed by the TGFBIp FAS1-4 domain, induced by corneal dystrophy–linked mutation V624M. Our work provides a framework for how high-resolution structures of protein fibrils can be harnessed to design end-capping peptide inhibitors, a procedure that may be applicable to disorders involving protein aggregation.

Corneal dystrophies are inherited, bilateral, noninflammatory disorders that present with corneal opacities of various shapes or loss of corneal transparency (1). Corneal dystrophy affects ~900 per million individuals in the United States (2). At present, the only treatment is keratoplasty, the transplant of donor cornea to replace the diseased cornea. However, keratoplasty requires precious healthy donor corneas which limits accessibility in certain parts of the world, involves potential complications such as graft failure or viral infection, and is only a temporary solution as disease recurrence is common 5 to 20 y post-operation (3–7). Alternative treatments are needed to improve accessibility and long-term patient outcome.

*TGFBI* gene, originally known as *βig-h3*, is widely expressed in human tissues (8) and the second most abundant protein in the corneal stroma (9, 10). TGFBIp is a 683-residue extracellular matrix protein composed of a cysteine-rich domain of periostin and TGFBIp (CROPT) domain at the N terminus, followed by four homologous fascilin 1 domains (FAS1-1 to FAS1-4) arranged like pearls on a string (11). Most human corneal TGFBIp is physiologically truncated at residue 657 (12). TGFBIp interacts with integrins (13) as well as structural extracellular matrix proteins including collagen (14) and fibronectin (15). It has been suggested to have roles in cell adhesion and migration, angiogenesis, wound healing, and inflammation (16); however its precise function is not known. In the late 1990s, autosomal dominant mutations in *TGFBI* were found to cause multiple corneal dystrophies (17–27). To date, there are 74 *TGFBI* mutations cataloged, wherein different mutations are associated with distinct corneal opacity phenotypes, disease presentations, and onsets (28–31). TGFBIp-linked corneal dystrophies include classic LCD (formerly known as type 1) and variants (formerly known as types III, IIIA, I/IIIA, IV, atypical LCD, and polymorphic corneal amyloidosis), GCD type 1 and type 2, Reis-Bücklers corneal dystrophy, and Thiel-Behnke corneal dystrophy (32). All LCD types are characterized by filamentous opacities in the stroma that stain positive for amyloid dye Congo Red (33, 34). GCD is defined by punctate deposits that contain Masson trichrome stained amorphous aggregates in type 1, and the presence of both amyloid and amorphous aggregates in type 2 (35–37).

Mass spectrometry analysis of corneal amyloid deposits from LCD patients with TGFBIp mutations V624M (38), A546D (39), A546D/P551Q (40), and H626R (41) identified enrichment of the same TGFBIp segment Y571-R588 from the FAS1-4 domain, relative to that detected in amorphous deposits from GCD patients and healthy donors. This segment forms protease-resistant amyloid fibrils in vitro (41, 42), further supporting its involvement in TGFBIp aggregation. Biochemical and in silico experiments suggest that mutations in FAS1-4, in which 62 of the 74 cataloged mutations are found (31), drive structural destabilization and indirectly cause amyloid formation (42–45). Currently, patient-derived TGFBIp aggregates have not been structurally characterized due to scarcity of patient tissues. A structural study of recombinant TGFBIp fibrils used a short peptide segment (46) instead of the globular FAS1-4 domain. Here, we determined a cryo-EM structure of fibrils formed by recombinant TGFBIp FAS1-4 proteins with LCD-linked mutation V624M.

## Results

### TGFBIp 563-657 Proteins Form Ribbon-Like Fibrils Readily.

We overexpressed and purified TGFBIp FAS1-4 domain (UniProtKB accession number Q15582, residues 503 to 657) proteins as well as a shorter construct of TGFBIp residues 563 to 657, which also contains the amyloidogenic Y571-R588 segment and we expected it to be more prone to aggregation. Proteins were cleaved of their N-terminal His6 and SUMO tags and subjected to affinity chromatography, so that pure TGFBIp monomer was used for all experiments (*SI Appendix*, Fig. S1). In addition to wild type (WT), we prepared proteins with LCD-linked mutations, particularly those carried by patients with the Y571-R588 segment enriched in their corneal amyloid deposits (38–41).

To initiate aggregation, we incubated TGFBIp 563-657 proteins at 30 µM in phosphate-buffered saline (PBS) pH 7.4 with intermittent shaking and monitored by ThT. Proteins with LCD-linked mutations G623R, V624M, and H626R, as well as WT proteins, form fibrils readily, completing their growth phase and reaching plateau around 16 h (*SI Appendix*, Fig. S2*A*). Negative stain transmission electron microscopy (nsTEM) at 16 h shows abundant fibrils for all proteins (*SI Appendix*, Fig. S2*B*). We collected cryo-EM data for TGFBIp 563-657 WT and V624M fibrils, however both fibrils exhibit ribbon-like morphology without regular twist, thus were unsuitable for structure determination by helical reconstruction (*SI Appendix*, Fig. S2 *C* and *D*). G623R and H626R fibrils appear similar in morphology under nsTEM and are possibly also ribbon-like without regular twist.

### TGFBIp FAS1-4 V624M Proteins Form Ribbon-Like and Twisting Fibrils.

**Fig. 1.**
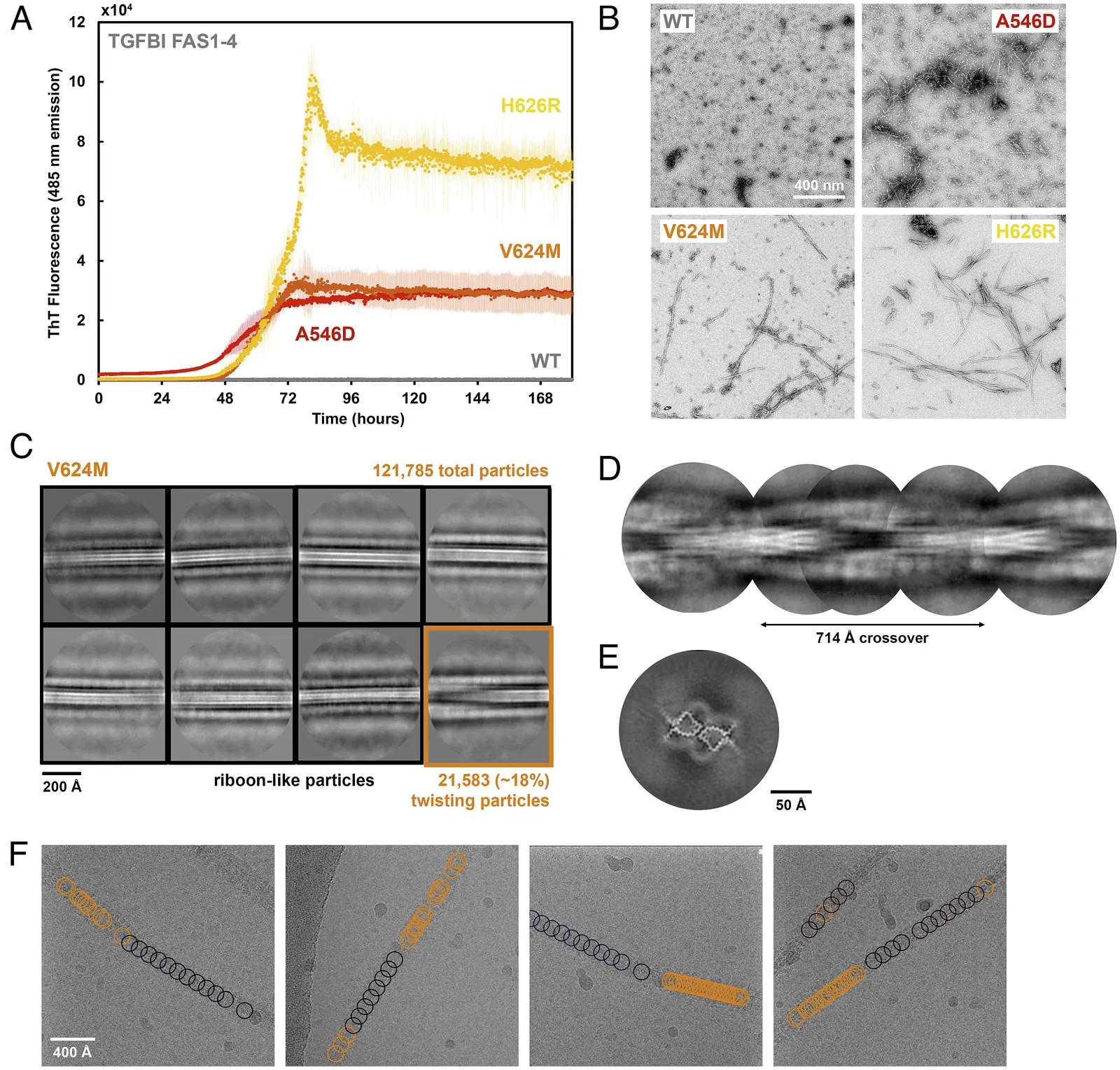
TGFBIp FAS1-4 V624M proteins form fibrils with ribbon-like and twisting morphology. (*A*) Thioflavin T kinetic assay of TGFBIp FAS1-4 (residues 503 to 657) proteins at 30 µm in PBS pH 7.4 with intermittent 700 rpm shaking. WT (gray) proteins and those with LCD-linked mutations A546D (red), V624M (orange), and H626R (yellow) were tested. (*B*) Negative stain transmission electron microscopy images of FAS1-4 fibrils at 6 d. (*C*) 2D classes from cryo-EM micrographs of FAS1-4 V624M fibrils exhibit ribbon-like (black boxes) and twisting (orange box) morphologies. (*D*) Stitched 2D classes of twisting fibrils. (*E*) 3D reconstruction of twisting fibrils with C2 symmetry. (*F*) Mapping of ribbon-like (black circles) and twisting (orange circles) particles onto cryo-EM micrographs.

Next, we incubated TGFBIp FAS1-4 proteins at 30 µM in PBS pH 7.4 with intermittent shaking and monitored by ThT. The FAS1-4 domain requires LCD-linked mutations A546D, V624M, or H626R to drive aggregation, completing their growth phase and reaching plateau around 3 d, whereas the WT protein does not aggregate even after 7 d (Fig. 1*A*). nsTEM at 6 d shows fibrils for FAS1-4 with LCD-linked mutations (Fig. 1*B*), sparse compared to 563-657 fibrils. Despite our attempts to structurally characterize FAS1-4 A546D and H626R fibrils as well, only FAS1-4 V624M fibrils were amenable for cryo-EM after 1 h treatment with 0.25% sodium dodecyl sulfate (*SI Appendix*, Fig. S3*A*). We collected cryo-EM data for FAS1-4 V624M fibrils, revealing mostly nontwisting ribbon-like fibrils and a minor population with twisting morphology (Fig. 1*C*). Twisting fibrils have a 714 Å crossover and C2 symmetry (Fig. 1*D*), and we resolved the ordered core by helical reconstruction (Fig. 1*E*). The structure of ribbon-like fibrils, the majority species, could not be determined. Particles from ribbon-like and twisting 2D classes were observed to originate from the same fibril (Fig. 1*F*), suggesting that ribbon-like fibrils could share the same structure as twisting fibrils (47).

### FAS1-4 V624M Amyloid Fibril Core Structure.

**Fig. 2.**
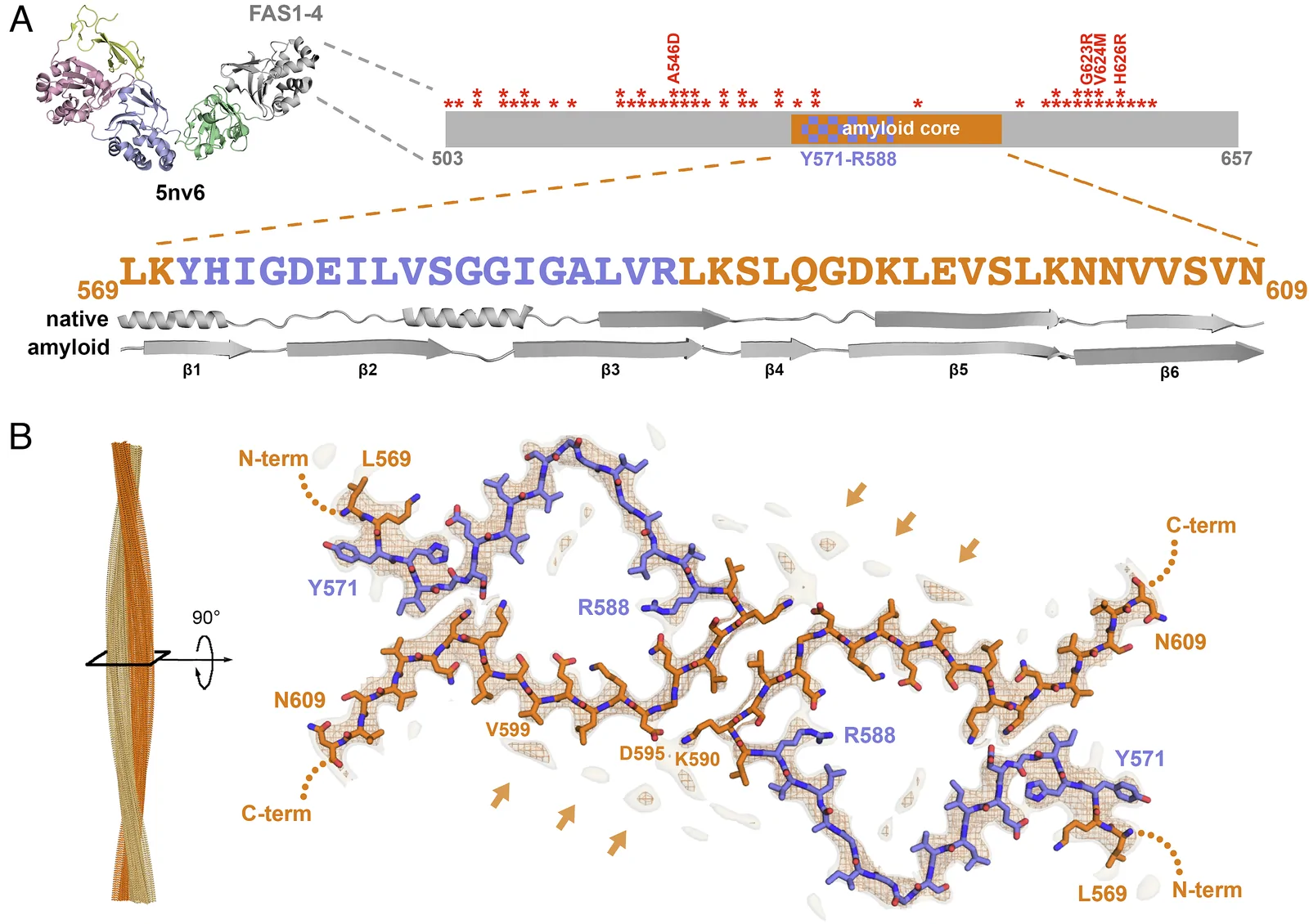
Cryo-EM structure of TGFBIp FAS1-4 V624M amyloid fibril core. (*A*) Cartoon model of the TGFBIp native structure (PDB 5nv6). FAS1-4 domain (gray) with annotations for corneal dystrophy–linked mutations (red asterisks), the L569 to N609 amyloid fibril core (orange) and the Y571-R588 segment (purple). Fibril core sequence aligned with native and amyloid secondary structures illustrated by helices, β-strands, and loops. (*B*) TGFBIp FAS1-4 V624M amyloid fibril reconstruction viewed perpendicular to the fibril axis (*Left*), sharpened cryo-EM map at high (orange mesh, 4.8 r.m.s.d.) and low (transparent surface, 3.4 r.m.s.d.) contour levels overlaid with refined atomic model of a cross-sectional layer (*Right*). The Y571-R588 segment (purple) is highlighted. Residual density near K590, D595, and V599 (arrows) may correspond to unordered C-terminal residues.

We determined a 3.2 Å cryo-EM structure of the twisting FAS1-4 V624M fibrils, with a helical twist of −1.21° and a helical rise of 4.8 Å (*SI Appendix*, Fig. S3 and Table 1). Out of the 155 residues of the FAS1-4 domain, 41 residues from L569 to N609 participate in the ordered fibril core. These residues arrange into a closed loop conformation resembling the Greek letter omega Ω, with twofold symmetric copies in each layer (Fig. 2). The Y571-R588 segment is in the core, while the V624M mutation is not. Strikingly, each protofilament has a prominent internal solvent cavity, which contrasts with most amyloids which are tightly packed to exclude water. The only mated β-strand is a homozipper at the protofilament dimer interface, featuring a hydrophobic interaction between two L592 residues. Minimal residual density near K590, D595, and V599 may be the C-terminal fuzzy coat wrapping back to interact with ordered core, also supported by weak density observed in the 3D class (Fig. 1*E*).

**Table 1.** Cryo-EM data collection, refinement, and validation statistics

Data collection and processing	Amyloid core	Attachments
Voltage (keV)	300	300
Magnification	165,000	165,000
Electron exposure (e^–^/Å^2^)	47.24	47.24
Defocus range (μm)	−0.5 to −3.5	−0.5 to −3.5
Pixel size (Å)	0.73	0.73
Symmetry imposed	C2	C2
Initial particle images	121,785	121,785
Final particle images	2,263	4,788
Helical rise (Å)	4.8	62
Helical twist (°)	−1.21	−15.63
Map resolution (Å)	3.24	15.2
FSC threshold	0.143	0.143
Map resolution range (Å)	3.24-200	15.2-200
Refinement		
Initial model used	De novo	
Model resolution (Å)	3.26	
FSC threshold	0.5	
Map sharpening B factor (Å^2^)	−19.7	
Model composition		
Nonhydrogen atoms	3070	
Protein residues	390	
Ligands	0	
B factors (Å^2^)		
Protein	93.8	
R.m.s. deviations		
Bond lengths (Å)	0.003	
Bond angles (°)	0.637	
Validation		
MolProbity score	2.56	
Clashscore	9.58	
Poor rotamers (%)	5.7	
Ramachandran plot		
Favored (%)	92.3	
Allowed (%)	7.7	
Disallowed (%)	0	

**Fig. 3.**
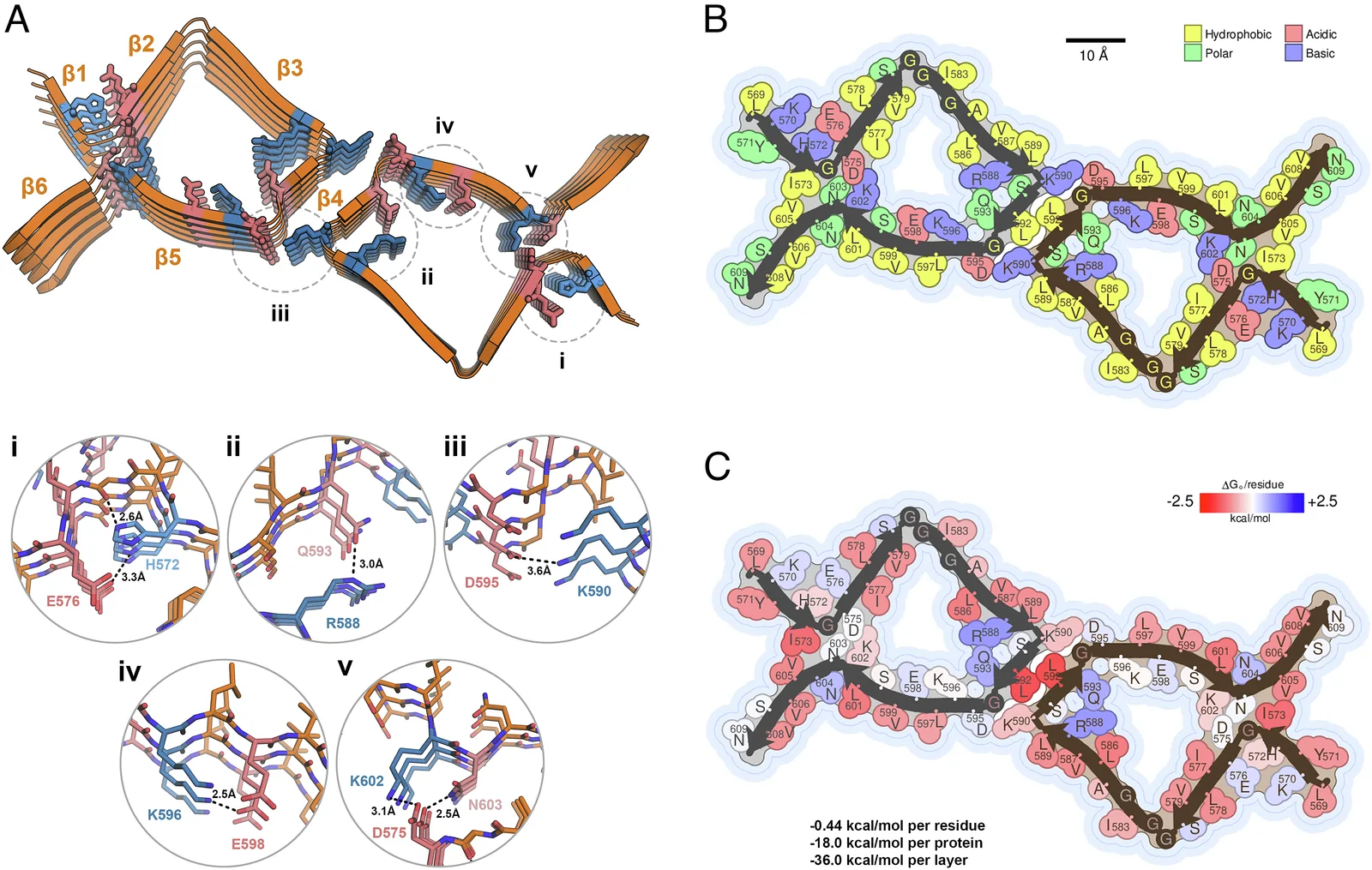
Electrostatic interactions, solvent-exposed hydrophobic β-strands, and stability evaluation of TGFBIp FAS1-4 V624M fibril core. (*A*) Cartoon model of five layers of TGFBIp FAS1-4 V624M fibril. β-strands of each protofilament are numbered β1 to β6. Electrostatic interactions between negatively charged (red), positively charged (blue), partially charged histidine (light blue), and polar (pink) residues are highlighted. Molecular details of contacts between i) H572, E576, and the G574 carbonyl, ii) R588 and Q593, iii) K590 and D595, iv) K596 and E598, and v) D575, K602, and N603. (*B*) Polarity map of TGFBIp FAS1-4 V624M fibril core. (*C*) Solvation energy map of TGFBIp FAS1-4 V624M fibril core with residues colored by atomic solvation standard free energy of stabilization.

The FAS1-4 V624M protofilament consists of six β-strands, β1 to β6 (Fig. 3*A*). Sequences in β3, β5, and β6 also form β-strands in the native FAS1-4 domain (Fig. 2*A*). Key electrostatic interactions coordinate the protofilament fold. H572, E576, and the G574 carbonyl (Fig. 3*A*i), as well as R588 and Q593 (Fig. 3*A*ii) mediate respective turns in the mainchain. Salt bridges between K590 and D595 (Fig. 3*A*iii) flank the hydrophobic contact in the dimer interface homozipper. D575, K602, and N603 (Fig. 3*A*v) help bring the protofilament loop to close. Notably, 8 of the 13 cavity-facing residues are polar or charged, possibly facilitating the passage of solvent molecules (Fig. 3*B*). Multiple hydrophobic β-strands are solvent-exposed, such as I577 to V579 on β2, a stretch of 8 residues G581 to L589 on β3, and L597 to L601 on the cavity-opposite side of β5 (Fig. 3*B*). However, these hydrophobic residues are partially buried by residues above and below along the fibril axis. This is reflected in the solvation energy map (Fig. 3*C*), in which hydrophobic residues score favorably and contribute to fibril stability. Overall, the atomic solvation standard free energy for the FAS1-4 V624M fibril is −0.44 kcal/mol per residue and −18.0 kcal/mol per protein, which is not as stable as pathogenic amyloids such as serum amyloid A1 (PDB 6mst, −34.4 kcal/mol per protein) (48) or tau CBD type II fibrils (PBD 6tjx, −38.0 kcal/mol per protein) (49), but more stable than functional amyloids such as orb2 (PBD 6vps, +0.8 kcal/mol per protein) (50) or RIPK1-RIPK3 fibrils (PDB 5v7z, −5.7 kcal/mol per protein) (51, 52).

### FAS1-4 V624M Fibril Attachments of Unknown Identity.

In cryo-EM micrographs, we observed FAS1-4 V624M fibrils decorated by globular attachments (*SI Appendix*, Fig. S4 *A* and *B*). We implemented a 20 Å high-resolution limit in 2D classification to avoid alignment to the 4.8 Å layers and estimated the averaged spacing of the attachments to be ~62 Å (*SI Appendix*, Fig. S4 *C* and *D*). Aiming to better resolve the attachments, we used a rise of 62 Å to refine attachment-bound twisting fibrils and obtained a 15 Å map (*SI Appendix*, Fig. S4 *E*–*G* and Table 1). The low resolution of the attachments, possibly due to heterogeneity or irregularity of bound molecules, prohibited their identification. Fibrils were formed with purified proteins (*SI Appendix*, Fig. S1*B*) in PBS without other components, thus attachments are most likely attributed to FAS1-4. We generated speculative models for the attachment density, including residues N-terminal to the amyloid core from adjacent layers associating as helical bundles predicted by AlphaFold3, as well as segments or monomers of FAS1-4 (*SI Appendix*, Fig. S5). These unidentified attachments may have an impact on the structure of the in vitro-formed fibrils.

### Structure-Based Design of Peptide Inhibitors.

**Fig. 4.**
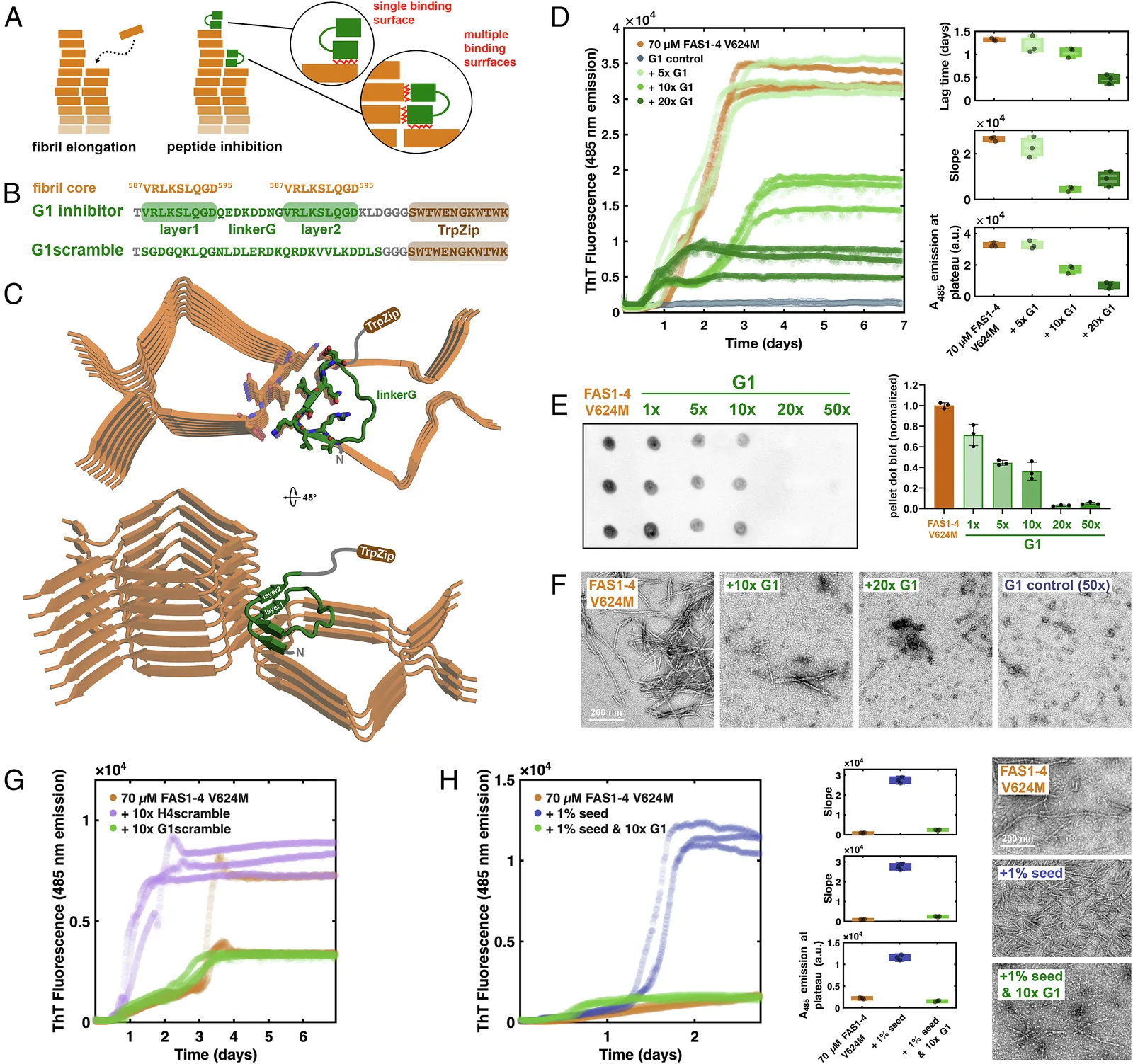
Structure-based design of G1 peptide inhibitor of TGFBIp FAS1-4 V624M fibril formation. (*A*) Schematic of peptide binding to TGFBIp fibril ends to inhibit elongation. (*B*) G1 peptide consists of layers 1 and 2 (green) which are homologous sequences to TGFBIp V587 to D595, Rosetta-optimized linkerG (green) and a tryptophan zipper (brown, TrpZip). (*C*) Cartoon model of G1 peptide (green) with TrpZip (brown) bound to end of FAS1-4 V624M fibril (orange) shown from two angles. G1 N terminus (N). (*D*) Thioflavin T kinetic assay of FAS1-4 V624M at 70 µm in PBS pH 7.4 with G1 at various concentrations, as well as G1 alone at 50× concentration (G1 control). Kinetic curve lag time, slope, and plateau fluorescence are quantified (*Right*). (*E*) TGFBIp antibody dot blot of the 100,000 g pellet of FAS1-4 V624M aggregation at 70 µm in PBS pH 7.4 with G1 at various concentrations. Dot blot intensities are quantified (*Right*). (*F*) Negative stain transmission electron microscopy images of FAS1-4 V624M aggregation with G1 at various concentrations, as well as G1 control at 50× concentration, at 7 d. (*G*) Thioflavin T kinetic assay of FAS1-4 V624M at 70 µm in PBS pH 7.4 with peptides of scrambled G1 and H4 sequences at 10x concentration. (*H*) Thioflavin T kinetic assay of FAS1-4 V624M at 70 µm in PBS pH 7.4 with fragmented FAS1-4 V624M fibrils (1% seed) and G1 at 10× concentration (*Left*). Kinetic curve lag time, slope, and plateau fluorescence are quantified (*Center*). Negative stain transmission electron microscopy images of FAS1-4 V624M aggregation with 1% seed and G1 at 10× concentration, at 3 d (*Right*).

We leveraged the FAS1-4 V624M fibril structure to design peptide inhibitors against TGFBIp aggregation. For our design, we used two repeats of the TGFBIp V587 to D595 fibril core segment located at the protofilament interface, intended to form two additional β-strand layers on top of homologous sequences at the fibril tip, producing three contact surfaces with the fibril (Fig. 4 *A*–*C*). The two layers are connected by a linker, which we optimized for length and sequence using Rosetta. A C-terminal tryptophan zipper was added to prevent inhibitor self-aggregation and enable absorbance detection during protein purification. Inhibitor binding is intended to obstruct incorporation of TGFBIp monomers into the fibril due to steric clash with the inhibitor linker and tryptophan zipper. We designed G1 and H4, with the same two V587 to D595 layers but different linker sequences, for synthesis and experimental testing (Fig. 4*B* and *SI Appendix*, Fig. S6*B*). We overexpressed and purified G1 and H4 peptides, cleaved and removed His6 and SUMO tags following a similar protocol for TGFBIp, so that pure G1 and H4 were used for inhibition experiments (*SI Appendix*, Fig. S6*A*).

To test the efficacy of our two inhibitors, we incubated FAS1-4 V624M at 70 µM in PBS pH 7.4 (conditions for cryo-EM sample) in the presence of G1 or H4 with intermittent shaking and monitored by ThT. G1 treatment reduces the overall fluorescence intensity and slope of kinetic curves in a concentration-dependent manner, however the lag time is shortened (Fig. 4*D*). After 7 d, we performed solubility fractionation in which aggregated TGFBIp was pelleted, quantified by dot blot, and observed to be reduced in a G1 concentration-dependent manner (Fig. 4*E*). Correspondingly, nsTEM shows reduction of fibrils in a G1 concentration-dependent manner (Fig. 4*F*). Similarly, H4 treatment reduces the fluorescence intensity and slope of ThT kinetic curves, aggregated TGFBIp quantified by dot blot, and fibrils in nsTEM in a concentration-dependent manner (*SI Appendix*, Fig. S6 *D*–*F*). Both G1 and H4 require 10 times the molar excess of TGFBIp or higher concentrations for notable inhibition effects. EGCG was used as a control (*SI Appendix*, Fig. S6*D*), previously shown to inhibit TGFBIp aggregation (53). Importantly, neither G1 nor H4 aggregate when incubated alone at the highest concentration for 7 d, as assessed by ThT and nsTEM. Peptides with scrambled G1 and H4 sequences did not exhibit inhibition, demonstrating the specificity of the designed peptides (Fig. 4 *B* and *G*).

We sonicated to fragment FAS1-4 V624M fibrils, generating “seeds” that can catalyze the conversion of monomers into fibrils and accelerate the kinetics of aggregation. As expected, FAS1-4 V624M fibril seeds increase the rate (slope) and totality (plateau ThT intensity) of monomer aggregation (Fig. 4*H*), suggesting that these TGFBIp fibrils possess the prion-like property of templated self-propagation. Addition of G1 reverses the effects of seed-induced aggregation (Fig. 4*H*), consistent with the idea that our peptide caps the ends of fibril seeds to block elongation. Finally, we tested whether G1 or H4, designed for the FAS1-4 V624M fibril structure, are effective on FAS1-4 aggregation driven by other LCD-linked mutations, A546D and H626R. Assessed by ThT and nsTEM, G1 and H4 require 20 times the molar excess of FAS1-4 A546D to significantly inhibit its aggregation (*SI Appendix*, Fig. S7 *A*–*C*). Conversely, G1 and H4 (the latter to a lesser degree) were effective at inhibiting FAS1-4 H626R starting at 5 times its molar excess, performing even better than on FAS1-4 V624M (*SI Appendix*, Fig. S7 *D*–*F*). These results suggest that FAS1-4 fibrils driven by different mutations could be targeted by the same peptide inhibitors to varying degrees, albeit all at high concentrations. TGFBIp with different mutations may share features in aggregation behavior or even structure, but ultimately require independent characterization.

## Discussion

Our molecular structure of the aggregated FAS1-4 domain of TGFBIp reveals an unanticipated solvent-penetrated amyloid fibril. Driven by LCD-linked mutation V624M, the formerly globular FAS1-4 domain flattens into 2-dimensional layers that stack on each other to form in-register β-strands arranged into an Ω-shaped fold. Each fibril layer consists of two copies of the Ω-shaped fold related by C2 symmetry, resulting in a cross-section that resembles a bowtie. The fibril core sequence, residues L569 to N609, includes the Y571-R588 segment found in LCD patient corneal deposits (38–41), supporting the potential disease-relevance of this structure. We note four caveats to consider for our FAS1-4 V624M fibril. First, our recombinant fibril may differ in structure from patient-derived fibrils. It remains to be seen whether in vitro-assembled and ex vivo TGFBIp fibrils have any resemblance, and whether different LCD-linked mutations yield similar or divergent folds. Second, the structure we determined is derived from the minority of fibrils or segments of fibrils which possess the requisite twist for helical reconstruction. We hypothesize that the majority ribbon-like species exhibits a similar fold to the twisting species, owing to the fact that twisting and ribbon-like segments are physically connected and presumably constrained by dozens of backbone hydrogen bonds to adopt related folds; however the degree of relatedness is undetermined (Fig. 1*F*). Third, fibrils are decorated by pseudoperiodic, unidentified globular attachments and their effect on fibril formation and fold is unknown (*SI Appendix*, Figs. S4 and S5). Fourth, fibrils were treated with 0.25% sodium dodecyl sulfate for 1 h to remove proteinaceous clumps and enable cryo-EM imaging (*SI Appendix*, Fig. S3*A*), which could influence their morphology, polymorph distribution, and globular attachments.

The unconventional core of the FAS1-4 V624M fibril differs from previous amyloid fibrils in three major respects. First, the open core surrounds solvent-filled cavities ~20 Å in diameter. Second, the Ω-shaped fold lacks common steric zipper interactions between β-strands in amyloid fibrils. The lone steric zipper in the structure forms the interface between the two protofilaments. Third, the polarity of amino acid residues is the reverse of that seen in most amyloid fibrils (52, 54). In most fibrils, sidechains on the inner surfaces are hydrophobic and sidechains on the outer surface are hydrophilic. In this structure, most sidechains exposed to the internal cavities are charged or polar (Lys, Arg, Asp, Glu, Gln, Ser) and sidechains on the outer surface are mainly hydrophobic (Leu, Ile, Val, Ala) (Fig. 3*B*). Here, the FAS1-4 V624M fibril represents an intriguing outlier to the norms of amyloid structure.

The pattern of LCD-linked mutations suggests that pathology is driven by destabilization of native structure. This hypothesis has been raised by the Enghild and Otzen groups and supported by biophysical comparison of wild-type TGFBIp proteins and those with corneal dystrophy–associated mutations (42–45). In one study, dynamic light scattering revealed that LCD-linked mutations, including V624M and H626R also featured in our study, caused FAS1-4 proteins to increase in hydrodynamic radii, suggestive of unfolding and oligomer formation (45). In our aggregation assays, LCD-linked mutations induce aggregation of FAS1-4 proteins while wild-type FAS1-4 remain inert after agitation for 7 d (Fig. 1 *A* and *B*). Many LCD-linked mutations, including the ones in this study, introduce bulkier or charged residues that are disruptive to the native structure (*SI Appendix*, Fig. S8), promoting unfolding and potential exposure of aggregation-prone segments. Thus, it is not surprising that the shorter TGFBIp construct (residues 563 to 657), likely with unprotected aggregation-prone segments, aggregates readily even without a mutation (*SI Appendix*, Fig. S2 *A* and *B*). For this reason, TGFBIp aggregation studies using the entire FAS1-4 domain driven by LCD-linked mutations may better recapitulate the native unfolding and amyloid folding pathway in disease. Notably, the FAS1-4 V624M fibril core is mostly devoid of LCD-linked point mutations (Fig. 2*A*), suggesting that mutations do not necessarily participate in amyloid formation and raising the possibility that different LCD-linked mutations yield convergent fibril folds. Taken together, these observations support the idea that the pathogenic role of LCD-linked mutations is native fold disruption, rather than amyloid fold stabilization.

Structure-based peptide inhibitors bind onto the ends of amyloid fibrils and cap their elongation, by preventing addition of monomers. Previously, we designed L- and D-peptide inhibitors against various fibril-forming proteins including tau (55, 56), amyloid-β (57, 58), α-synuclein (59), transthyretin (60), islet amyloid protein (57, 61), immunoglobulin light chain (62), and SARS-CoV-2 nucleocapsid protein (63). While the potential of peptides for treating neurodegenerative diseases is challenged by delivery into patient brains, this issue is not present for ocular diseases. Here, we implemented a design strategy that takes advantage of symmetric protofilaments. Extension of the two protofilaments is likely not synchronized, so that one protofilament will generally be some layers longer than the other. This allows for an inhibitor peptide to adhere via multiple interfaces at the fibril tip—the layer below on the shorter protofilament and the layers across on the longer protofilament—increasing its binding affinity (Fig. 4*A*). Experimentally, G1 and H4 inhibitors reduce FAS1-4 fibril formation driven by multiple LCD mutations in a concentration-dependent manner quantified by ThT fluorescence, solubility fractionation, and nsTEM (Fig. 4 and *SI Appendix*, Fig. S6). G1 nullifies the amplified aggregation caused by addition of fibril seeds (Fig. 4*H*). Taken together, these results indicate that G1 and H4 impede TGFBIp fibril elongation, consistent with our design rationale. However, the high molar excess required for their efficacy is unexpected for a primarily fibril end capping mechanism, since the number of fibril ends should be substoichiometric to the number of TGFBIp proteins after aggregation is initiated. Thus, other mechanisms may be involved, such as sequestration of monomers, modulation of primary or secondary nucleation, stabilization of off-pathway species, or altered fibril fragmentation (64). In particular, we note the peptides shorten the lag time of the ThT kinetic curves. These peptides, which contain segments homologous to the fibril core, may reduce the energy barrier for nucleation or stabilize early-stage oligomers. G1 and H4 hindered TGFBIp aggregation our tested in vitro conditions, however they have not been evaluated in cell and animal models. Furthermore, TGFBIp aggregation inhibitors with clinical aspirations should be designed using disease-relevant structures when those become available.

In conclusion, our cryo-EM structure of FAS1-4 V624M fibrils reveals initial molecular details of TGFBIp amyloid aggregation, in the absence of patient-derived structures. We provide proof of concept for structure-based inhibitors as a potential interventional strategy for corneal dystrophy and other ocular diseases involving protein aggregation.

## Methods

### Protein Overexpression and Purification.

DNA gene blocks (Integrated DNA Technologies) of bacteria codon-optimized TGFBIp (UniProtKB accession number Q15582) residues 502 to 682 with N-terminal His6 and SUMO tags, as well as designed and scrambled G1 and H4 peptide inhibitors with N-terminal His6 and SUMO tags, were subcloned into pET28a(+) expression plasmid via NcoI and SacI restriction enzyme sites. Truncation and LCD-linked mutation constructs were generated using standard gene splicing by overlap extension protocol (65) and confirmed by sequencing (Plasmidsaurus). Plasmids were transformed into BL21(de3) or BL21(de3) Gold Competent Cells (Agilent). Bacterial cultures were grown in LB Broth at 37 °C with 200 rpm shaking until 0.8 to 1.0 optical density at 600 nm, then protein overexpression was induced with 1 mM isopropyl β-D-1-thiogalactopyranoside. After 4 to 5 h, cells were harvested by centrifugation at 8,000 g at 4 °C for 10 min, and pellets were flash frozen in liquid nitrogen and stored at −80 °C. Cell pellets were thawed on ice, resuspended in Loading Buffer (50 mM HEPES pH 7.5, 150 mM NaCl), lysed using an Ultrasonic Vibra Cell Processor (Sonics & Materials Inc.) at 80% power with 3 s on–off cycles for 4 min, centrifuged at 33,000 g at 4 °C for 40 min, and the supernatant was filtered using 0.22 μm Polyethersulfone Membrane Filters (Thermo Fisher Scientific). Immobilized metal affinity chromatography (IMAC) was performed using 5 mL HisTrap HP columns (Cytiva) operated by an NGC Chromatography System (Bio-Rad). Filtered cell lysate was loaded onto IMAC columns equilibrated in Loading Buffer, washed with ten column volumes of Loading Buffer with 20 mM imidazole, and proteins were eluted by an Elution Buffer (50 mM HEPES pH 7.5, 150 mM NaCl, 1 M imidazole) gradient of 1%/mL. Purified proteins were buffer exchanged into 50 mM Tris-HCl pH 7.6 and 100 mM NaCl, then incubated with homemade His-tagged Ulp1 protease at 1:100 Ulp1:protein concentration and 1 mM dithiothreitol at 30 °C for 2 h. His6/SUMO-cleaved proteins were purified by IMAC, in which the flowthrough was collected. Proteins were subjected to gel electrophoresis using 4 to 12% NuPAGE Bis-Tris Mini Protein Gels (Thermo Fisher Scientific) to confirm purity and Ulp1 cleavage. Purified His6/SUMO-cleaved proteins were concentrated using Amicon Ultra-15 Centrifugal Filters (MilliporeSigma), flash frozen in liquid nitrogen and stored at −80 °C.

### Thioflavin T Kinetic Assay.

TGFBIp proteins were diluted to 30 µM in phosphate-buffered saline pH 7.4 with 40 µM Thioflavin T. For assays with peptide inhibitors, FAS1-4 proteins were diluted to 70 µM in phosphate-buffered saline pH 7.4 with 80 µM Thioflavin T, and various concentrations of G1 or H4 were added: 70 µM (1×), 350 µM (5×), 700 µM (10×), 1.4 mM (20×), 3.5 mM (50×). Preformed FAS1-4 V624M fibrils were sonicated using a Vibra Cell VC500 (Sonics & Materials) in a water bath in pulsed mode (50% duty cycle, level 9 output control) for 5 min to produce short fibril fragments, confirmed by negative stain transmission electron microscopy. 1% (v/v) fibril fragments was added for seeding experiments. Samples were aliquoted into 100 µL or 200 µL triplicates in 96 Well Black/Clear Bottom Plates (Thermo Fisher Scientific) with a 32 mm polytetrafluoroethylene bead (Saint Gobain Performance Plastics) in each well. For each condition, a well without Thioflavin T was prepared for negative stain transmission electron microscopy. Samples were incubated in a FLUOstar Omega microplate reader (BMG Labtech) at 37 °C with 700 rpm shaking for 30 s every 5 min. Thioflavin T fluorescence was measured every 5 min with 448 nm excitation and 482 nm emission. Kinetic assay plots were illustrated using MATLAB.

### Negative Stain Transmission Electron Microscopy.

Formar/Carbon Support Films on 400 Mesh Copper Grids (Ted Pella, Inc.) were glow discharged for 30 s. 3 µL of TGFBIp sample was applied to grids, incubated for 2 min, then excess solution was blotted off. 3 µL of 2% uranyl acetate (Electron Microscopy Sciences) was applied to the grids, incubated for 2 min, then excess solution was blotted off. Another 3 µL of 2% uranyl acetate was applied to the grids, excess solution was blotted off immediately, then the grids were air dried. Grids were imaged using a Tecnai T12 Transmission Electron Microscope (FEI).

### Fibril Formation for Cryo-EM.

TGFBIp 563-657 WT and V624M proteins were diluted to 30 µM in phosphate-buffered saline pH 7.4, then incubated in a FLUOstar Omega microplate reader at 37 °C with 700 rpm shaking for 30 s every 5 min. Fibrils were harvested after 16 h for cryo-EM. To remove nonfibril proteins, samples were centrifuged at 164,000 g for 30 min and the pellet was resuspended in equal volume of distilled water. TGFBIp FAS1-4 V624M proteins were diluted to 70 µm in phosphate-buffered saline pH 7.4, then incubated in a Digital Orbital Mixing Chilling/Heating Dry Baths (Torrey Pines Scientific) with 900 rpm shaking at 37 °C. Fibrils were harvested after 6 d for cryo-EM. To declump fibrils, samples were incubated with 0.25% sodium dodecyl sulfate for 1 h, then cryo-EM grids were prepared immediately.

### Cryo-EM.

Quantifoil R1.2/1.3 Holey Carbon Films on 200 Mesh Copper Grids (Electron Microscopy Sciences) were glow discharged for 2 min. 3 µL of fibril sample was applied to the grids, which were then blotted and plunge frozen in liquid ethane at 100% humidity using a Vitrobot Mark IV System (Thermo Fisher Scientific). Grids were imaged using a Titan Krios Transmission Electron Microscope (Thermo Fisher Scientific) equipped with a Falcon 4i Direct Electron Detector (Thermo Fisher Scientific), operated at 300 keV voltage with 20 eV energy filter. Data collection was performed using Leginon (66).

### Helical Reconstruction.

Motion correction of raw movie frames was performed using MotionCorr (67) implemented in RELION-4.0 (68). Contrast transfer function estimation was performed using CTFFIND4 (69). Particles were picked manually using e2helixboxer.py from EMAN2 (70), which were then used to train automated particle picking using crYOLO (71, 72). Particle extraction, 2D classification, helical reconstruction, and 3D refinement were performed using RELION-4.0 (73, 74). Particles were extracted with 32-pixel interbox using 640-pixel box size binned by two, or 320-pixel box size. For TGFBIp FAS1-4 V624M fibrils, 2D classification revealed a majority of ribbon-like fibrils and a minor twisting polymorph. Particles from 2D classes of twisting fibrils were selected for initial helical reconstruction with a reference model generated de novo using those 2D class averages. Multiple rounds of 3D classification were performed by manually controlling the tau2_fudge and healpix_order parameters, poor particles were discarded and helical refinement was performed on the optimized subset of particles. Contrast transfer function refinement, Bayesian polishing, and golden-standard 3D autorefinement were performed. The final map was sharpened using phenix.auto_sharpen (75). For fibril attachments, particles were extracted with 32-pixel interbox using 480-pixel box size. 2D classification was performed with a 20 Å high-resolution limit. Particles from 2D classes of twisting fibrils with resolved attachments were selected for helical reconstruction using 62 Å rise and -15.63 twist (calculated from the crossover distance of the amyloid core, 714 Å). Golden-standard 3D autorefinement was performed and the final map was sharpened using phenix.auto_sharpen (75). Cryo-EM data collection and processing statistics are in Table 1.

### Atomic Modeling and Refinement.

TGFBIp FAS1-4 V624M amyloid fibril core atomic model was built and adjusted using Coot (76), with iterative refinement using phenix.real_space_refine (77). Model validation was performed using phenix.comprehensive_validation (78) and MolProbity (79). Map and model visualization, molecular analysis, and generation of “strain” scores for rotamers were performed using PyMOL (80). Model composition and validation statistics are in Table 1. For fibril attachments, residues N-terminal to the amyloid core were modeled due to their relative proximity. With considerations for linker distance from fibril core and approximate size of attachment density, FAS1-4 sequences and number of copies were submitted for AlphaFold3 (81) prediction. Output models were manually inspected, docked into attachment density, scored for CC_mask_ using Phenix (77), and the best models were shown.

### Peptide Inhibitor Design.

From a 10-layer model of the FAS1-4 V624M fibril, 5 layers from one protofilament were removed except V587 to D595 of two layers above the remaining 5 layers, defined as the inhibitor. RosettaRemodel (82) was used to optimize length and sequence of the linker between the two layers via a *blueprint* file that specified fixed backbone for the two layers and residue sampling for the linker. 10,000 iterations were performed for each linker length, sorted by total score calculated by weighted energy terms, and top models were manually inspected using PyMOL.

### Solubility Fractionation.

Thioflavin T kinetic assay endpoint samples were centrifuged at 100,000 g for 20 min. The supernatant was carefully extracted and the pellet was resuspended in equal volume of phosphate-buffered saline pH 7.4. 2 μL of sample was pipetted three times onto 0.2 μm Nitrocellulose Membranes (Bio-Rad), then air-dried. Membranes were blocked in 5% (w/v) Blocking-Grade Blocker (Bio-Rad) in Tris buffered saline with 0.1% (v/v) Tween20 (TBST) for at 22 °C for 1 h or at 4 °C overnight, incubated with 1:1,000 dilution of TGFBI Antibody (Novus Biologicals, Catalog # NBP1-60049, Lot # QC14319-20141010) in 2% (w/v) Blocking-Grade Blocker in TBST for 1 h, washed three times with TBST, incubated with 1:4,000 dilution of Goat Anti-Rabbit Secondary Antibody HRP (Thermo Fisher Scientific, Catalog # 31460) in 2% (w/v) Blocking-Grade Blocker in TBST for 1 h, washed three times with TBST, incubated with Pierce ECL Plus Western Blotting Substrate (Thermo Fisher Scientific), and imaged using an Azure Imaging System (Azure Biosystems).

## Supplementary Material

Appendix 01 (PDF)

## Data Availability

Cryo-EM micrographs were deposited in Electron Microscopy Public Image Archive (EMPIAR) with accession code EMPIAR-13341 (83). Cryo-EM maps were deposited in Electron Microscopy Data Bank (EMDB) for TGFBIp FAS1-4 V624M amyloid fibril core with accession code EMD-70400 (84) and for fibril attachments with accession code EMD-70401 (85). Atomic model of FAS1-4 V624M amyloid fibril core was deposited in Protein Data Bank (PDB) with accession code 9oek (86). All other data are included in the manuscript and/or *SI Appendix*.
