# Supplementary material for: Cryo-EM structure of TGFBIp fibrils driven by a corneal dystrophy–linked mutation enables design of peptide inhibitors of aggregation
Source: Proc Natl Acad Sci U S A. 2026 Sep 8;123(37):e2607937123. doi: 10.1073/pnas.2607937123 (PMC13578946; doi:10.1073/pnas.2607937123)
Supplement: Supplementary file 1 [file pnas.2607937123.sapp.pdf]

PNAS

**Supporting Information for**

# Cryo-EM structure of TGFBIp fibril driven by a corneal dystrophy-linked mutation enables design of peptide inhibitors of aggregation.

Yi Xiao Jiang[1]*, Lukasz Salwinski[1], Michael R. Sawaya[1], Peng Ge[1], Liisa Lutter[1], Xinyi Cheng[1], Carolyn J. Hu[1], Hillary Hernandez[1], David R. Boyer[1], Conrad Wang[1], Filipe A. Melo[1], Duilio Cascio[1], David S. Eisenberg[1]*.

[1] Department of Chemistry and Biochemistry, Department of Biological Chemistry, UCLA-DOE Institute, and Molecular Biology Institute, UCLA, Los Angeles, CA, 90095, USA.

*Corresponding Authors: Yi Xiao Jiang and David S. Eisenberg
Email: seanjiang@ucla.edu and david@mbi.ucla.edu

**This PDF file includes:**

Figures S1 to S8

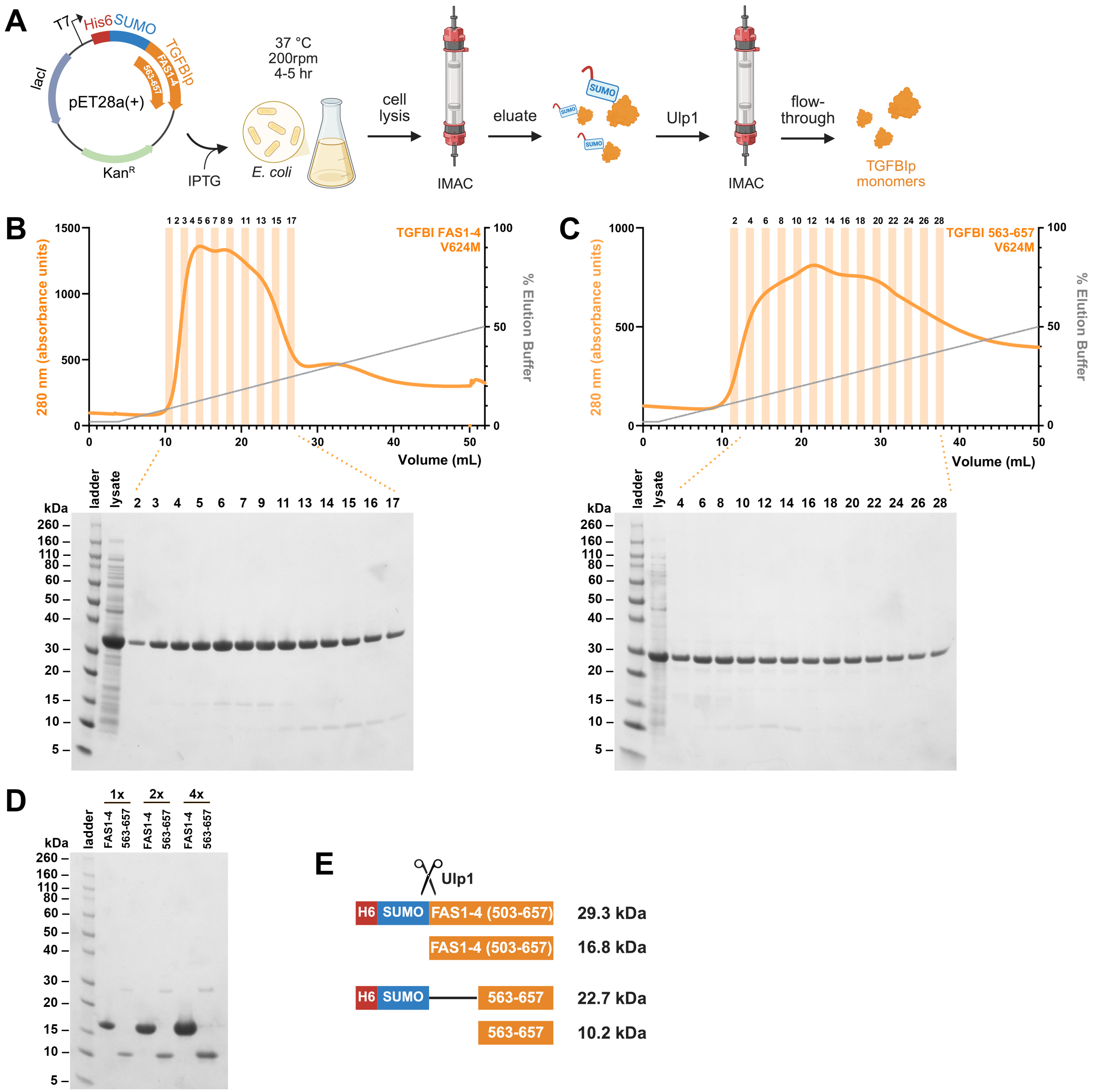


**Fig. S1. TGFBIp overexpression and purification. A)** Schematic of TGFBIp overexpression and purification. **B,C)** Immobilized metal affinity chromatography (IMAC) chromatogram of TGFBIp FAS1-4 V624M (B) and TGFBIp 563-657 V624M (C) elution using imidazole gradient (top) and SDS-PAGE gel of collected fractions (bottom). **D)** SDS-PAGE gel of TGFBp FAS1-4 and 563-657 V624M after Ulp1 cleavage of His6 and SUMO tags and IMAC purification. **E)** TGFBIp constructs and molecular weights.

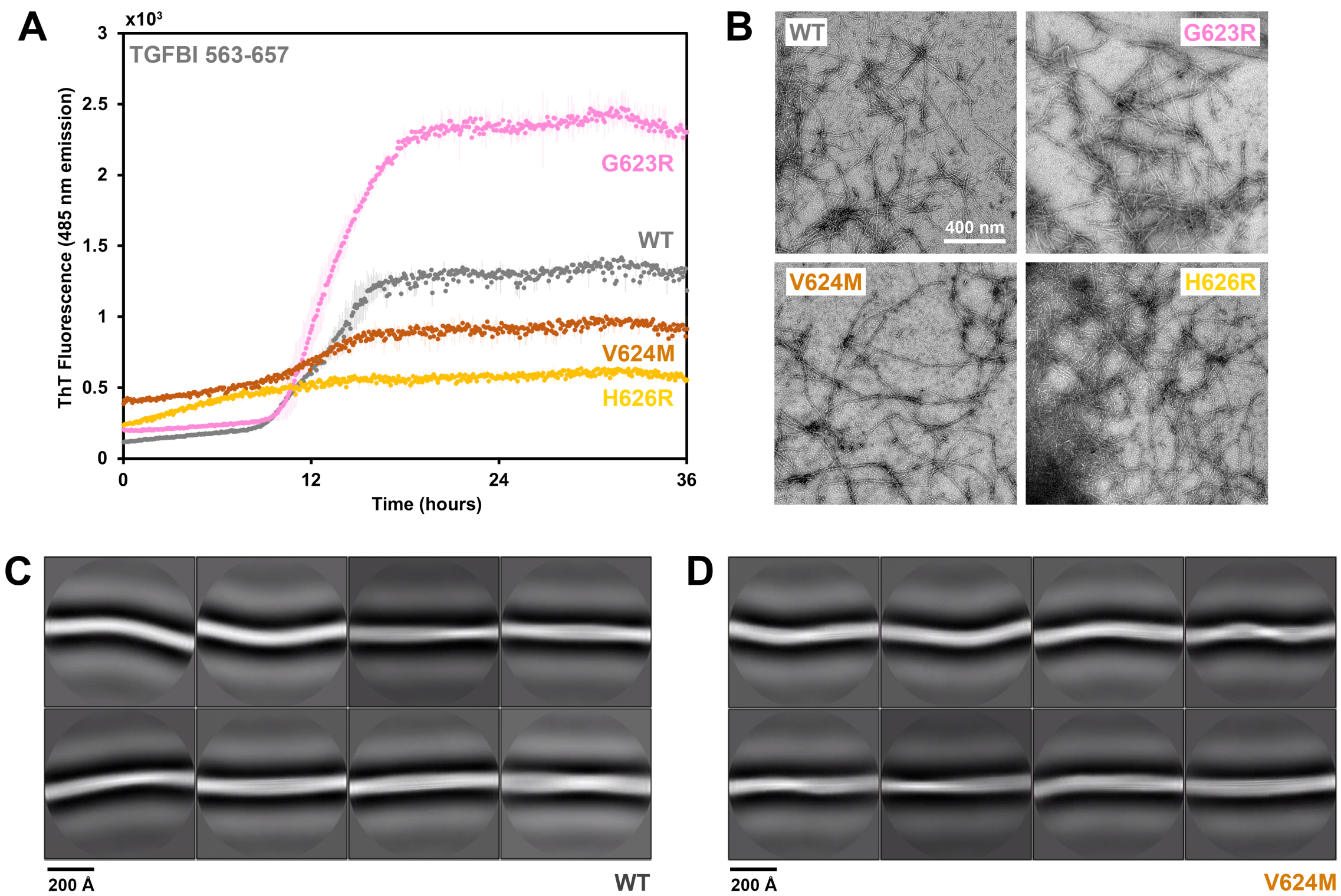


**Fig. S2. TGFBIp 563-657 proteins form ribbon-like fibrils readily. A)** Thioflavin T kinetic assay of TGFBIp 563-657 proteins at 30 µm in PBS pH 7.4 with intermittent 700rpm shaking. WT (gray) proteins and those with LCD-linked mutations G623R (pink), V624M (orange) and H626R (yellow) were tested. **B)** Negative stain transmission electron microscopy images of TGFBIp 563-657 fibrils at 16 hours. **C,D)** 2D classes from cryo-EM micrographs of TGFBIp 563-657 WT (C) and V624M (D) fibrils exhibit ribbon-like morphology without regular twist 60-100 Å in diameter.

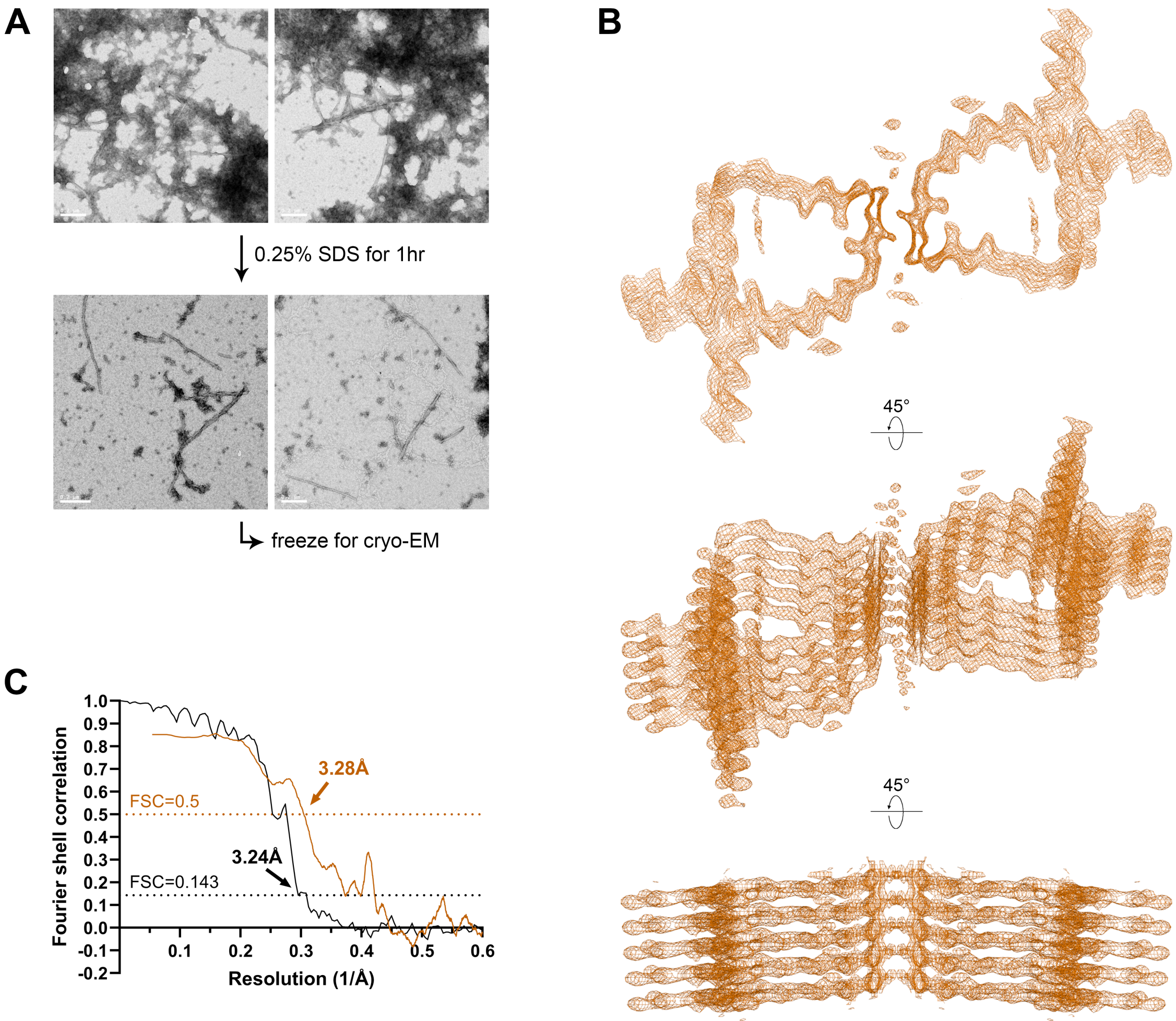


**Fig. S3. Cryo-EM of TGFBIp FAS1-4 V624M fibrils. A)** Negative stain transmission electron microscopy images of TGFBIp FAS1-4 V624M fibrils before and after 0.25% sodium dodecyl sulfate treatment for 1 hour. **B)** Five layers of TGFBIp FAS1-4 V624M fibril core cryo-EM map shown from various angles. **C)** Fourier shell correlation (FSC) curves for two independent half-maps (black) and the refined atomic model against the cryo-EM map (orange). FSC thresholds 0.143 (black dashed line) and 0.5 (orange dashed line).

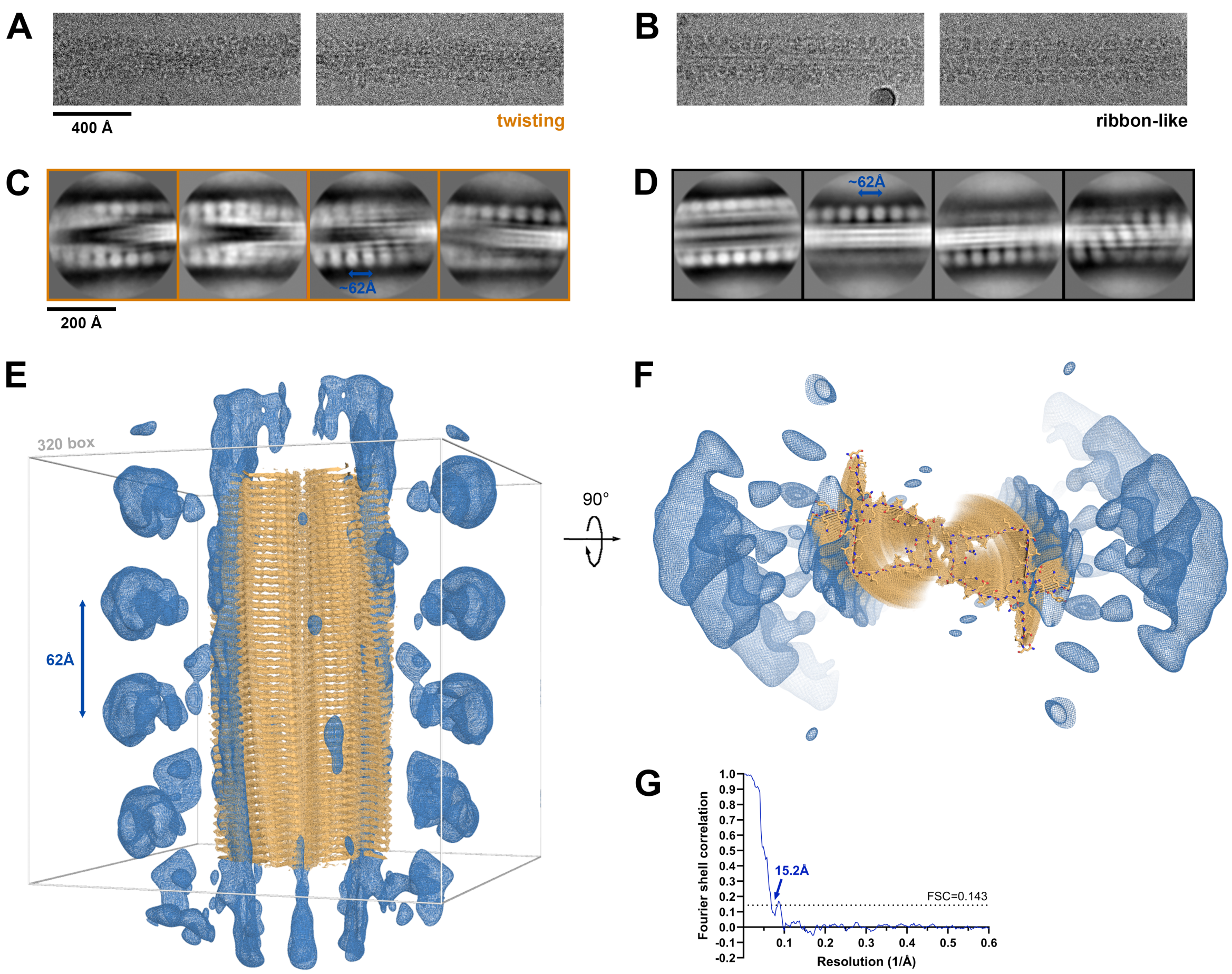


**Fig. S4. TGFBIp FAS1-4 V624M fibrils are decorated by attachments of unknown identity. A,B)** Cryo-EM micrographs of attachment-bound twisting (A) and ribbon-like FAS1-4 V624M fibrils (B). **C,D)** 2D classes from twisting (C) and ribbon-like FAS1-4 V624M fibrils (D). Attachments were resolved by implementing a 20 Å high resolution limit. **E,F)** Cryo-EM map of FAS1-4 V624M fibril attachments (blue mesh, 4.0 r.m.s.d.) obtained by helical reconstruction of 480 box size particles with 62 Å rise. FAS1-4 V624M fibril core cartoon model and cryo-EM map from 320 box size particles (orange) are overlaid. Side (E) and top views down the fibril axis (F) are shown. **G)** Fourier shell correlation (FSC) curve for two independent half-maps (blue) of FAS1-4 V624M fibril attachments. FSC threshold 0.143 (black dashed line).

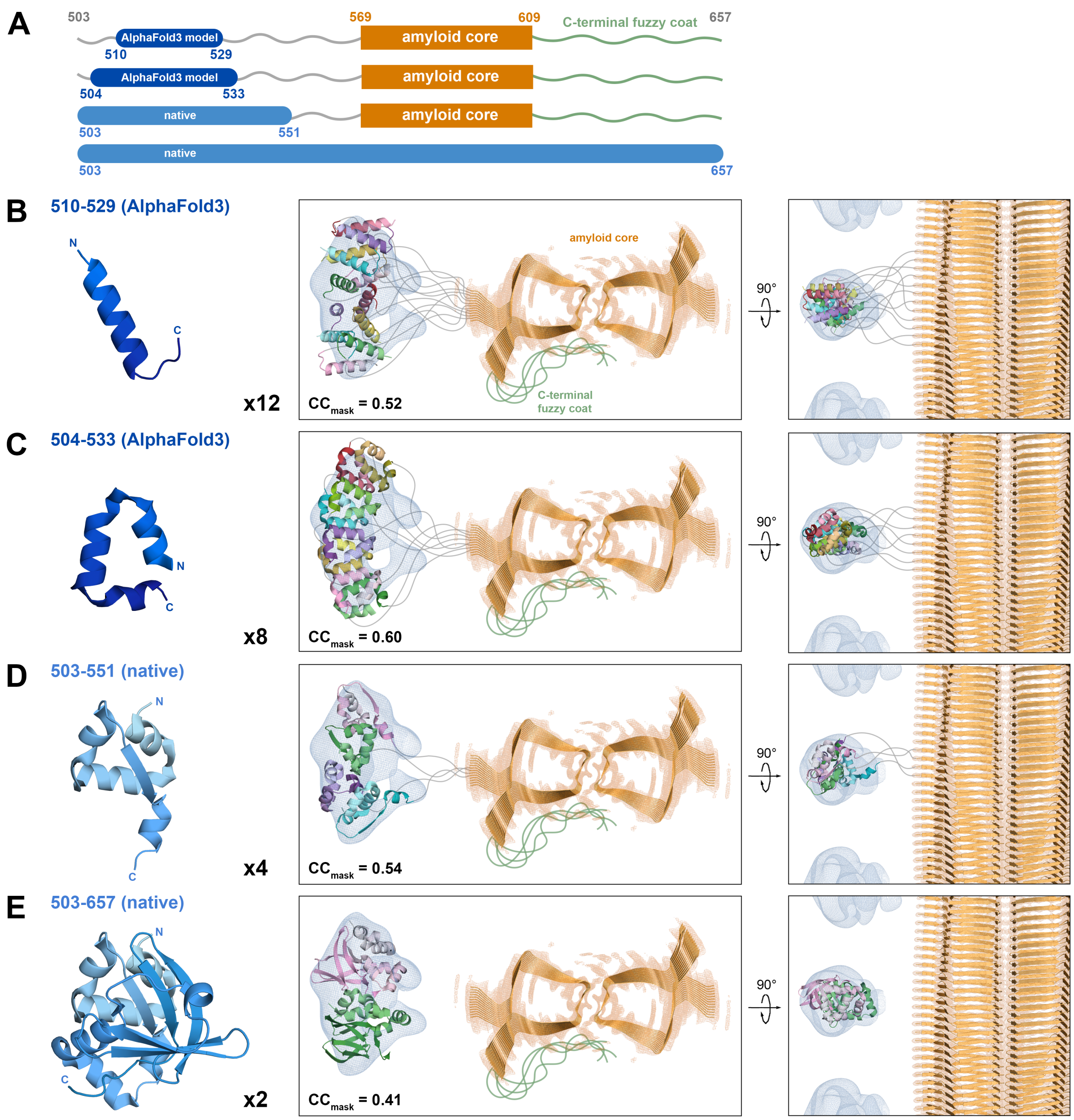


**Fig. S5. Speculative models for TGFBIp FAS1-4 V624M fibril attachments. A)** Summary of FAS1-4 residues contributing to FAS1-4 V624M fibril core (orange), fibril attachments (dark or light blue), and fuzzy coat (green) for the proposed models. **B)** Residues 510-529 N-terminal to the fibril core from 12 adjacent layers form a helix bundle, modeled by AlphaFold3. **C)** Residues 504-533 N-terminal to the fibril core from 8 adjacent layers form a helix bundle, modeled by AlphaFold3. **D)** Residues 503-551 N-terminal to the fibril core from 4 adjacent layers form a tetramer in their native fold. **E)** FAS1-4 monomers dimerize and bind to fibril side. For B,C,D,E, cartoon models of fibril attachment subunits (left), top (center) and side views (right) of cartoon models and cryo-EM maps of the fibril core (orange) and fibril attachments (blue) are shown. $CC_{mask}$ indicates model-to-map correlation.

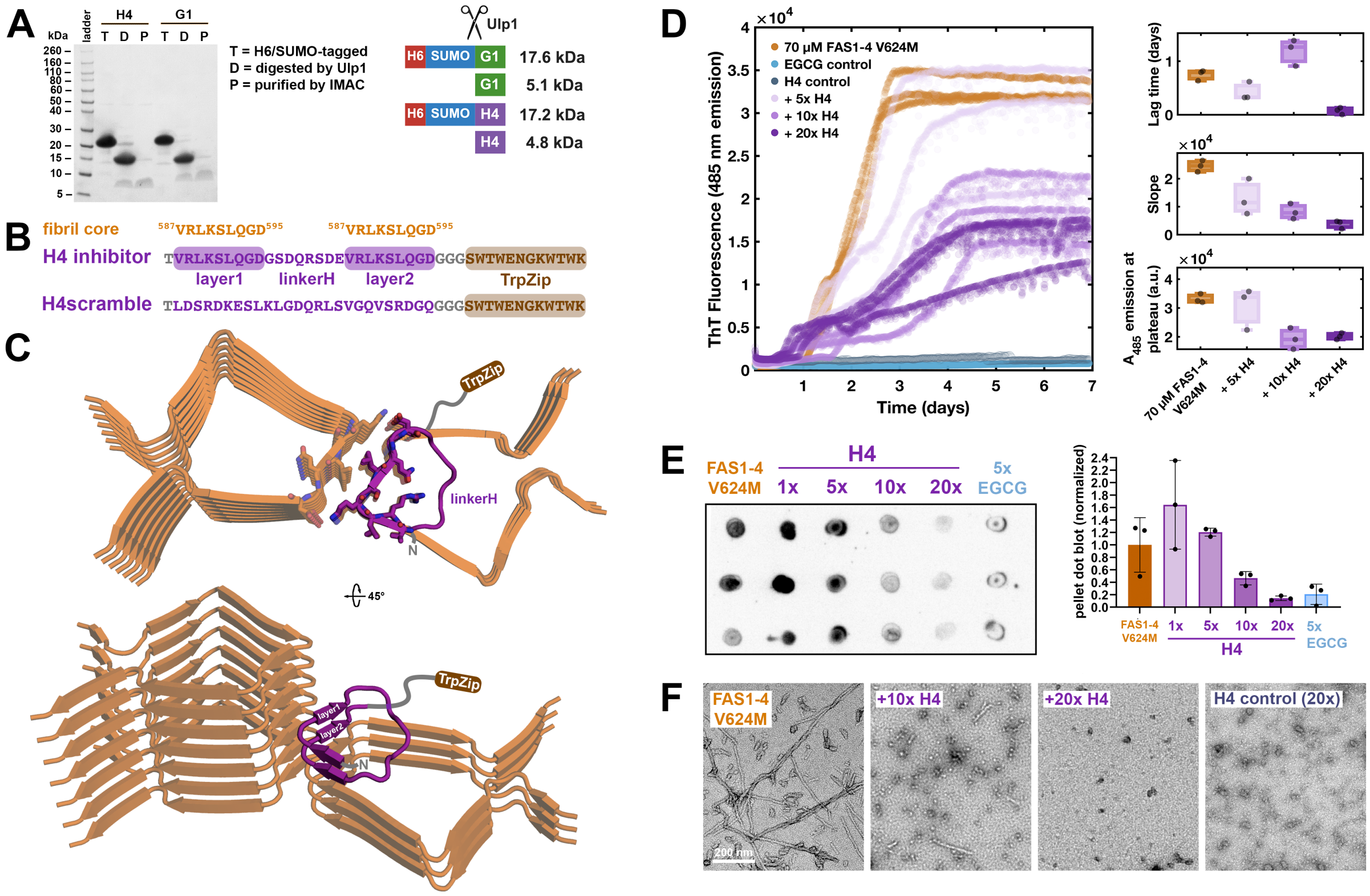

**Fig. S6. Structure-based design of H4 peptide inhibitor of TGFBIp FAS1-4 V624M fibril formation. A)** SDS-PAGE gel of G1 and H4 peptides as purified H6/SUMO-tagged proteins (T), digested by Ulp1 (D), then purified by IMAC (P). G1 and H4 constructs and molecular weights (right). **B)** H4 peptide consists of layers 1 and 2 (purple) which are homologous sequences to TGFBIp V587 to D595, Rosetta-optimized linkerH (purple) and a tryptophan zipper (brown, TrpZip). **C)** Cartoon model of H4 peptide (purple) with TrpZip (brown) bound to end of FAS1-4 V624M fibril (orange) shown from two angles. H4 N-terminus (N). **D)** Thioflavin T kinetic assay of FAS1-4 V624M at 70 µm in PBS pH 7.4 with epigallocatechin gallate at 350 µm (5x EGCG control, light blue) or H4 at various concentrations, as well as H4 alone at 20x concentration (H4 control). Kinetic curve lag time, slope, and plateau fluorescence are quantified (right). **E)** TGFBIp antibody dot blot of the 100,000 *g* pellet of FAS1-4 V624M aggregation at 70 µm in PBS pH 7.4 with 5x EGCG or H4 at various concentrations. Dot blot intensities are quantified (right). **F)** Negative stain transmission electron microscopy images of FAS1-4 V624M aggregation with H4 at various concentrations, as well as H4 control at 20x concentration, at 7 days.

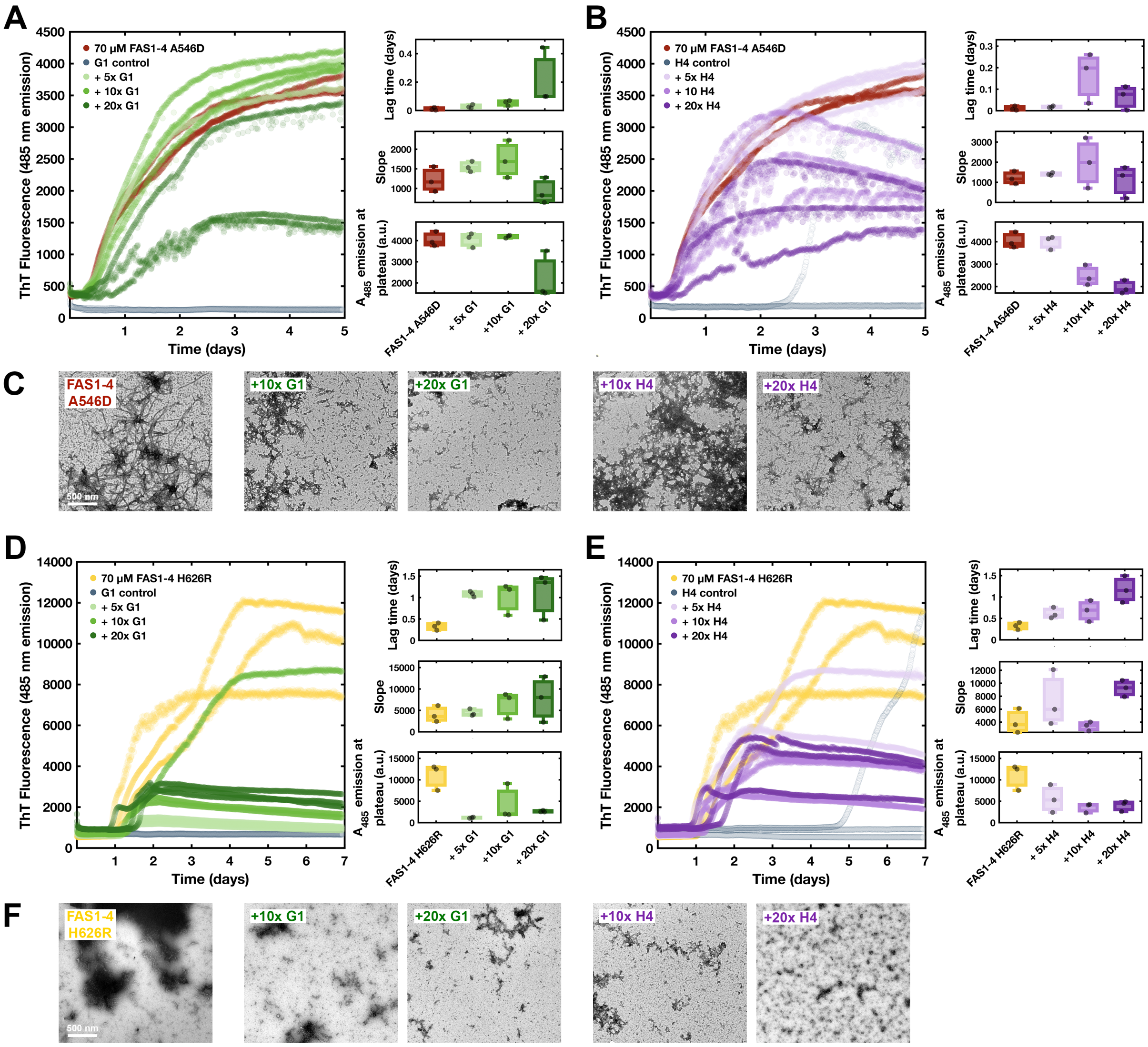


**Fig. S7. G1 and H4 peptides inhibit TGFBIp FAS1-4 A546D and H626R fibril formation. A,B)** Thioflavin T kinetic assays of FAS1-4 A546D at 70 µm in PBS pH 7.4 with G1 (A) or H4 (B) at various concentrations, as well as G1 or H4 alone (control). Kinetic curve lag time, slope, and plateau fluorescence are quantified. **C)** Negative stain transmission electron microscopy images of FAS1-4 A546D aggregation with G1 or H4 at various concentrations at 5 days. **D,E)** Thioflavin T kinetic assays of FAS1-4 H626R at 70 µm in PBS pH 7.4 with G1 (D) or H4 (E) at various concentrations, as well as G1 or H4 alone (control). Kinetic curve lag time, slope, and plateau fluorescence are quantified. **F)** Negative stain transmission electron microscopy images of FAS1-4 H626R aggregation with G1 or H4 at various concentrations at 7 days.

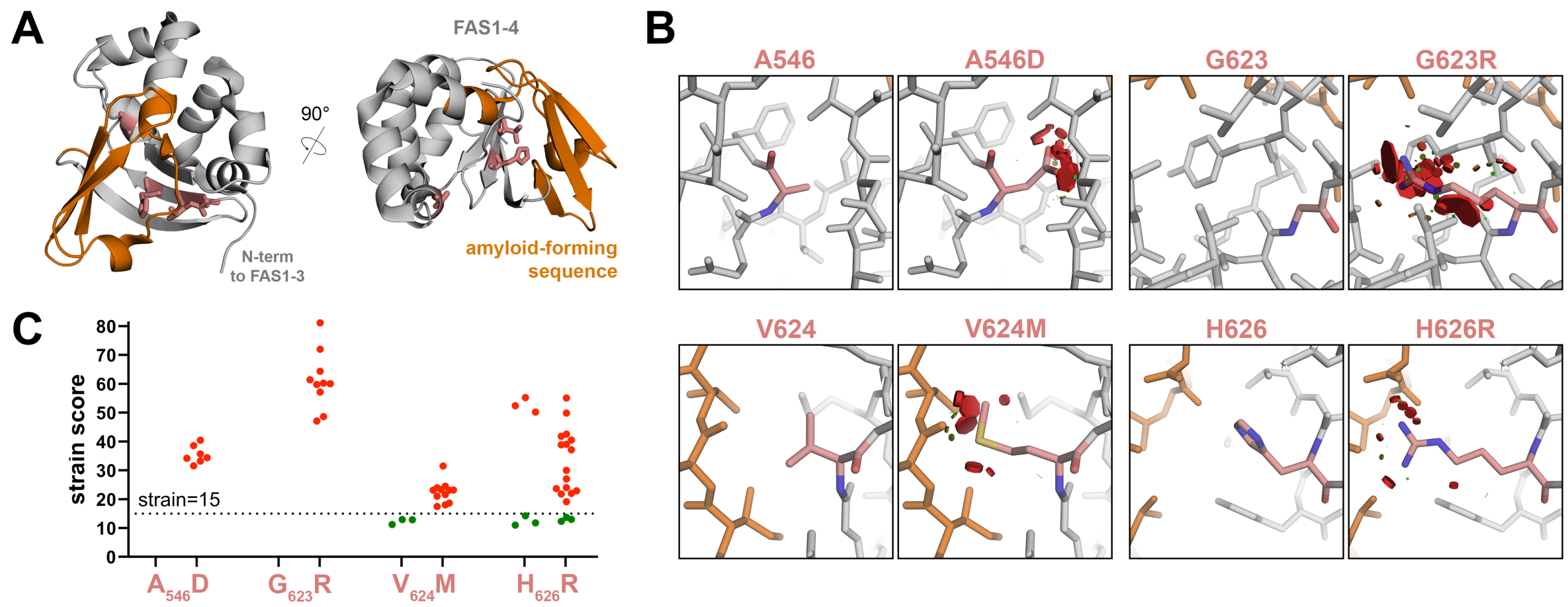


**Fig. S8. Lattice corneal dystrophy-linked mutations destabilize the TGFBIp native fold. A)** Native structure of TGFBIp FAS1-4 domain (PDB 5nv6) with fibril-forming sequences (orange) highlighted. Residues of LCD-linked mutations A546D, G623R, V624M and H626R are shown as sticks (pink). **B)** Comparison of wildtype and mutation residues (pink) in the native structure. Red disks indicate van der Waals overlap between atoms. **C)** Strain scores generated by PyMOL's Mutagenesis Wizard for each rotamer (dot) of wildtype and mutation residues in the native structure. Strain scores higher than 15 (red dots) indicate significant van der Waals overlap and rotamer incompatibility. Strain scores for alanine and glycine, with single rotamers, are not available.
